# Predicting in-hospital death during acute presentation with pulmonary embolism to facilitate early discharge and outpatient management

**DOI:** 10.1371/journal.pone.0179755

**Published:** 2017-07-13

**Authors:** Jerrett K. Lau, Vincent Chow, Alex Brown, Leonard Kritharides, Austin C. C. Ng

**Affiliations:** 1 Department of Cardiology, Concord Hospital, University of Sydney, Sydney, New South Wales, Australia; 2 South Australian Health and Medical Research Institute, Adelaide, South Australia, Australia; Azienda Ospedaliero Universitaria Careggi, ITALY

## Abstract

**Background:**

Pulmonary embolism continues to be a significant cause of death. The aim was to derive and validate a risk prediction model for in-hospital death after acute pulmonary embolism to identify low risk patients suitable for outpatient management.

**Methods:**

A confirmed acute pulmonary embolism database of 1,426 consecutive patients admitted to a tertiary-center (2000–2012) was analyzed, with odd and even years as derivation and validation cohorts respectively. Risk stratification for in-hospital death was performed using multivariable logistic-regression modelling. Models were compared using receiver-operating characteristic-curve and decision curve analyses.

**Results:**

In-hospital mortality was 3.6% in the derivation cohort (n = 693). Adding day-1 sodium and bicarbonate to simplified Pulmonary Embolism Severity Index (sPESI) significantly increased the C-statistic for predicting in-hospital death (0.71 to 0.86, P = 0.001). The validation cohort yielded similar results (n = 733, C-statistic 0.85). The new model was associated with a net reclassification improvement of 0.613, and an integrated discrimination improvement of 0.067. The new model also increased the C-statistic for predicting 30-day mortality compared to sPESI alone (0.74 to 0.83, P = 0.002). Decision curve analysis demonstrated superior clinical benefit with the use of the new model to guide admission for pulmonary embolism, resulting in 43 fewer admissions per 100 presentations based on a risk threshold for admission of 2%.

**Conclusions:**

A risk model incorporating sodium, bicarbonate, and the sPESI provides accurate risk prediction of acute in-hospital mortality after pulmonary embolism. Our novel model identifies patients with pulmonary embolism who are at low risk and who may be suitable for outpatient management.

## Introduction

Venous thromboembolism is a common cardiovascular disease, with pulmonary embolism (PE) its most severe manifestation [1]. PE occurs in approximately 100 per 100,000 people annually [1]. The predictors of early mortality after PE include advanced age, medical comorbidities and clinical features at presentation [2–5].

The Pulmonary Embolism Severity Index (PESI) [6] and the simplified Pulmonary Embolism Severity Index (sPESI) [7] are validated prediction scores to risk stratify patients hospitalized with acute PE. Calculated based on patient’s demographics, comorbidities, and initial clinical assessment findings, they have been shown to predict 30-day mortality. The PESI and sPESI however, do not incorporate any biomarkers. In addition, there are currently no risk models to predict in-hospital death after acute PE.

Hyponatremia has been shown to be a predictor of in-hospital and 30-day all-cause mortality after acute PE across all strata of PESI risk [8, 9]. Arterial base deficit has also been reported to be a marker of PE severity [10].

The present study investigates the potential for low serum sodium and low serum bicarbonate to contribute to risk stratification in acute PE, which may be used to identify low risk patients suitable for outpatient management. We demonstrate the addition of day-1 serum sodium and bicarbonate values to the sPESI significantly improves in-hospital mortality risk stratification after acute PE.

## Materials and methods

### Study population

Since January 2000, all consecutive patients admitted to Concord Hospital (Sydney, New South Wales, Australia) with a confirmed principal diagnosis of PE have been entered into a PE database. The outcomes of patients from this database have been reported previously [8, 11, 12]. Patients admitted between January 2000 and December 2012 were identified with non-local state residents excluded in order to minimize incomplete tracking of outcomes. All patients had their diagnosis of PE confirmed as per published guidelines [13]. For patients with recurrent PE, only their initial presentation was included in the study.

### Data collection

Data variables extracted from medical record were entered into the PE database by trained medical personnel (V.C. and A.N.). Data variables collected included details of patient’s admission and comorbidities (see Text A in S1 File for details of variables collected). Chronic kidney disease was defined as an estimated glomerular filtration rate <60mL/min/1.73m^2^. Values outside the institution laboratory reference ranges were regarded as abnormal.

The sPESI score was calculated based on age (>80years), history of malignancy, chronic cardiopulmonary disease, heart rate ≥110beats/min, systolic blood pressure ≤100mmHg, and oxyhemoglobin saturation <90% [7].

### Study outcomes

The primary outcome was in-hospital all-cause mortality. In-hospital mortality was chosen to facilitate development of a model to determine if patients can be managed in an outpatient setting from day-1. A state-wide death registry database was used to verify and determine the cause of death. Each death certificate was reviewed independently by at least two reviewers (J.L., L.K. or A.N.), with disparities resolved by consensus. The cause of death was coded in accordance with the World Health Organization guideline [14].

### Derivation and validation cohorts

Patients presenting in the odd years between 2001 and 2011 constituted the derivation cohort and were used to develop the new predictive model for in-hospital mortality after acute PE. To determine the applicability of the new model, we validated it in patients who presented in the even years between 2000 and 2012.

### Statistical analysis

Continuous variables are expressed as means plus-minus standard deviations (SD), while categorical variables are presented as numbers and frequency percentage. Comparisons between continuous variables were performed using the unpaired t test or the Mann-Whitney U test. Categorical data were compared using χ^2^ tests or Fisher’s exact test.

To determine the predictors of in-hospital death, univariable and multivariable logistic regression analyses were performed. Univariable parameters assessed included age, gender, sPESI, other comorbidities not accounted for in sPESI, serum sodium and bicarbonate values. Univariables with P<0.1 were included in the multivariable modelling analysis. Only predictors with a correlation coefficient ≤0.7 with either sPESI, sodium or bicarbonate were included. The area under the receiver operating characteristic (ROC) curve (AUC or C-statistic) was used to assess the discrimination performance of each model in predicting in-hospital death and these were compared using the DeLong test [15]. The prognostic performance of adding sodium and bicarbonate to sPESI were examined using net reclassification (four-risk category model) and integrated discrimination improvement with sodium and bicarbonate analyzed both as continuous and as dichotomous variables, with optimum levels derived from the Youden index [16]. The validation data were not accessed until the final model, derived in the derivation data, was selected and fixed. This model was the only model evaluated in the validation cohort.

A decision curve analysis was performed to compare the clinical usefulness and net benefits of model 1 (sPESI) and model 2 (sPESI + sodium + bicarbonate) with regards to the risk of in-hospital death [17]. The benefits of these models were compared to the clinical practice of either admitting all patients or discharging all patients.

We performed two additional analyses to assess the validity of our study. Multiple imputations (20 imputations) were used to account for missing data [18]. Secondly, we performed a population-linkage analysis to verify the mortality outcome of our PE cohort was comparable to the rest of the state (New South Wales) population admitted with acute PE within the same study time period. A censored date of 31 December 2013 was pre-defined for the linkage analysis of all-cause mortality. Cox proportional hazards regression analysis was used to compare survival curves adjusted for age and sex.

In order to compare the performance of the derived model with the original sPESI, the performance of each model in predicting 30-day mortality of our total PE patient cohort was separately analyzed using multivariable modelling and ROC curve analyses as described above.

All analyses were performed on de-identified data using SPSS v22 (IBM, USA) or Stata v14.1 (StataCorp LP, USA). A two-tailed probability value <0.05 was considered statistically significant.

### Ethical considerations

The study protocol conforms to the ethical guidelines of the 1975 Declaration of Helsinki. The involved institutional committees granted ethics approval: Concord Hospital PE cohort (CH62/6/2008-009) and population-linkage analysis (2013/09/479). The committees also granted a waiver of the requirement for consent from the individual for use of their health information. All patient data was de-identified and analyzed anonymously.

## Results

The derivation cohort consisted of 693 patients, with mean age 67.3±16.5years and 44.7% male patients. A history of cardiovascular disease, malignancy, and deep vein thrombosis was present in 39.0%, 22.1% and 18.8% of patients respectively. The mean sPESI on admission was 0.9±0.9. Day-1 serum sodium and bicarbonate levels were recorded in 95.7% and 95.8% of patients with mean values of 138.6±3.9 mmol/L and 24.6±3.7 mmol/L respectively. Low sodium (<135 mmol/L) and low bicarbonate (<24 mmol/L) were identified in 13.1% and 40.2% of patients respectively. None of these parameters differed significantly between the derivation and validation cohorts (Table 1).

**Table 1:** Baseline characteristics of derivation cohort and validation cohort*.

Characteristic	Derivation (n = 693)	Validation (n = 733)
Age, years	67.3 ± 16.5	67.8 ± 16.3
Male, no. (%)	310 (44.7)	319 (43.5)
CVD, no. (%)	270 (39.0)	276 (37.7)
Peripheral vascular disease, no. (%)	72 (10.4)	71 (9.7)
Stroke, no. (%)	20 (2.9)	20 (2.7)
Hypertension, no. (%)	180 (26.0)	174 (23.7)
Diabetes, no. (%)	91 (13.1)	100 (13.6)
Dyslipidemia, no. (%)	78 (11.3)	74 (10.1)
Current smoking, no. (%)	58 (8.4)	59 (8.0)
CRD, no. (%)	79 (11.4)	95 (13.0)
Pulmonary hypertension, no. (%)	11 (1.6)	13 (1.8)
DVT during admission, no. (%)	130 (18.8)	154 (21.0)
Malignancy, no. (%)	153 (22.1)	148 (20.2)
Chronic kidney disease, no. (%)	44 (6.3)	35 (4.8)
**Hemodynamic and biochemistry parameters on admission**		
Systolic blood pressure, mmHg†	140.2 ± 24.0	140.0 ± 25.0
Heart rate, beats/min‡	87.9 ± 21.1	88.6 ± 21.2
Oxyhemoglobin saturation, %§	95.6 ± 3.7	95.3 ± 4.6
sPESIǁ	0.9 ± 0.9	0.9 ± 0.9
Day-1 Na<135mmol/L, no. (%)	87/663 (13.1)	80/715 (11.2)
Day-1 Na, mmol/L	138.6 ± 3.9	138.6 ± 4.0
Day-1 HCO3<24mmol/L, no. (%)	267/664 (40.2)	301/714 (42.2)
Day-1 HCO3, mmol/L	24.6 ± 3.7	24.3 ± 3.5
Day-1 eGFR, mL/min/1.73m^2^¶	78.7 ± 34.9	77.0 ± 29.3
**Outcome**		
In-hospital death, no. (%)	25 (3.6)	20 (2.7)

* Plus-minus values are means ± standard deviation. There were no significant differences between the two groups.

^†^ Number of patients with admission systolic blood pressure recorded in the derivation and validation cohorts were 630/693 and 676/733 respectively.

^‡^ Number of patients with admission heart rate recorded in the derivation and validation cohorts were 630/693 and 676/733 respectively.

^§^ Number of patients with admission oxyhemoglobin saturations recorded in the derivation and validation cohorts were 610/693 and 646/733 respectively.

^ǁ^ Number of patients with sPESI calculated in the derivation and validation cohorts were 610/693 and 646/733 respectively.

^¶^ Number of patients with admission eGFR recorded in the derivation and validation cohorts were 662/693 and 713/733 respectively.

CVD, cardiovascular disease (included coronary artery disease, heart failure, valvular heart disease and arrhythmias); CRD, chronic respiratory disease (included asthma, chronic obstructive pulmonary disease and interstitial lung disease); DVT, deep vein thrombosis; eGFR, estimated glomerular filtration rate; HCO3, serum bicarbonate; Na, serum sodium; sPESI, simplified Pulmonary Embolism Severity Index; The sPESI incorporates age >80 years, history of malignancy, chronic cardiopulmonary disease, heart rate ≥110 beats/minute, systolic blood pressure <100 mmHg and oxyhemoglobin saturation <90%.

Patients with low sodium were older, more likely to have pre-existing cardiovascular disease, prior stroke, and malignancy, and more likely to die in-hospital than those with sodium ≥135 mmol/L (Table A in S1 File). In contrast, there were no significant differences between patients with low bicarbonate and those with bicarbonate ≥24mmol/L with respect to baseline comorbidities. There was weak evidence of increased in-hospital death in those with low bicarbonate (5.2% vs 2.5%, P = 0.09) (Table B in S1 File).

The rates of in-hospital death did not differ across the 13 years of the registry (P = 0.81) or between the derivation and validation cohorts (3.6% versus 2.7% respectively, P = 0.37). Of the 25 patients in the derivation cohort and 20 patients in the validation cohort who died in-hospital, 22 (88%) and 13 (65%) died due to PE. The rates of in-hospital death due to PE did not differ significantly between the derivation and validation cohorts (P = 0.08).

### Predictors of in-hospital death—Derivation cohort

Univariable predictors of in-hospital death included older age, increasing sPESI score, lower sodium and bicarbonate levels. Multivariable analysis demonstrated that in-hospital death was independently associated with sPESI (odds ratio [OR] per-1-point, 1.75; 95% confidence interval [CI] 1.13–2.70), sodium (OR per-1 mmol/L increase, 0.83; 95% CI 0.76–0.90), and bicarbonate (OR per-1 mmol/L increase, 0.87; 95% CI 0.77–0.98) (Table 2).

**Table 2:** Predictors of in-hospital death after acute PE (derivation cohort).

Variables	Odds ratio (95% CI)	P value
**Univariable analysis**		
Age, per 1-year increase	1.03 (1.00–1.06)	0.04
Male	1.35 (0.61–3.01)	0.46
sPESI, per 1-point increase	2.06 (1.39–3.04)	<0.001
Valvular heart disease	1.95 (0.25–15.4)	0.53
Atrial fibrillation	1.53 (0.56–4.17)	0.41
Hypertension	0.70 (0.26–1.91)	0.49
Diabetes	0.87 (0.26–3.07)	0.87
Dyslipidemia	0.32 (0.04–2.40)	0.27
Current smoking	0.45 (0.06–3.36)	0.43
Day-1 Na, per 1mmol/L increase	0.81 (0.74–0.88)	<0.001
eGFR, per 1mL/min/1.73m^2^ increase	0.99 (0.98–1.01)	0.40
Day-1 HCO3, per 1mmol/L increase	0.84 (0.74–0.94)	0.004
Multivariable analysis*		
sPESI, per 1-point increase	1.75 (1.13–2.70)	0.01
Day-1 Na, per 1mmol/L increase	0.83 (0.76–0.90)	<0.001
Day-1 HCO3, per 1mmol/L increase	0.87 (0.77–0.98)	0.03

* Age was not included in the multivariable logistic regression analysis as age is a variable used to calculate the sPESI.

CI, confidence interval; HCO3, serum bicarbonate; Na, serum sodium; sPESI, simplified Pulmonary Embolism Severity Index.

The sPESI incorporates age >80 years, history of malignancy, chronic cardiopulmonary disease, heart rate ≥110 beats/minute, systolic blood pressure <100 mmHg and oxyhemoglobin saturation <90%.

**Fig 1:**
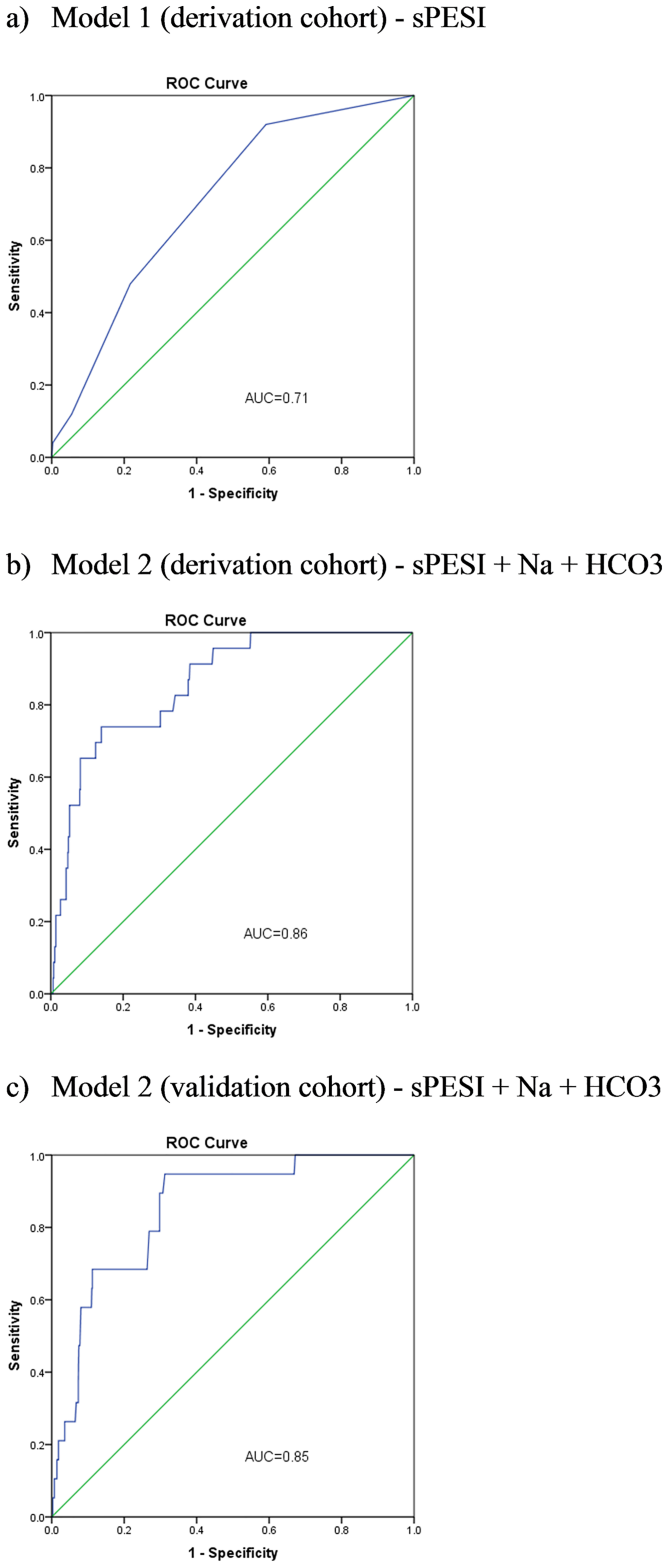
Impact of adding serum sodium and bicarbonate to sPESI for prediction of in-hospital mortality. The area under the ROC curve (AUC) for sPESI (model 1) (a) for predicting in-hospital death in the derivation cohort was 0.71 (95% CI 0.62–0.80). The AUC for the model sPESI + day-1 Na + day-1 HCO3 (model 2) (b) (Na and HCO3 as continuous variables) for predicting in-hospital death in the derivation cohort was 0.86 (95% CI 0.79–0.93). In the validation cohort the AUC for model 2 (c) was 0.85 (95% CI 0.78–0.92). sPESI, simplified Pulmonary Embolism Severity Index; Na, serum sodium; HCO3, serum bicarbonate; ROC, receiver operating characteristics. The sPESI incorporates age >80 years, history of malignancy, chronic cardiopulmonary disease, heart rate ≥110 beats/minute, systolic blood pressure <100 mmHg and oxyhemoglobin saturation <90%.

Fig 1a and 1b demonstrates the improvement associated with adding sodium and bicarbonate to sPESI (model 2) in predicting in-hospital death following acute PE in the derivation cohort. The AUC for sPESI (model 1) (AUC_sPESI_) was 0.71 (95% CI 0.62–0.80). Model 2 increased the AUC (AUC_sPESI+sodium+bicarbonate_) to 0.86 (95% CI 0.79–0.93) (AUC_sPESI_ versus AUC_sPESI+sodium+bicarbonate_, P = 0.001) for predicting in-hospital death (Table C in S1 File).

### Model validation

The validation cohort was comprised of 733 patients, with baseline characteristics presented in Table 1. Fig 1c shows model 2 ROC curve for predicting in-hospital death in the validation cohort. Model 2 C-statistics for the derivation and validation cohorts were similar at 0.86 (95% CI 0.79–0.93) and 0.85 (95% CI 0.78–0.92) respectively (Table C in S1 File).

### New model net reclassification performance

The addition of sodium and bicarbonate as continuous variables to sPESI was associated with a net reclassification improvement (NRI) for the derivation cohort estimated at 0.613 (P = 0.0007) and an integrated discrimination improvement (IDI) of 0.067 (P = 0.001) (Table 3). The event NRI was 0.39 and the non-event NRI was 0.22.

**Table 3:** Reclassification of patients (derivation cohort).

Established model -sPESI*	sPESI + day-1 serum sodium and bicarbonate*				
	<2% risk	2–5% risk	5–10% risk	≥10% risk	Total no.
Patients who died, no.					
<2% risk	0	0	2	0	2
2–5% risk	1	2	2	5	10
5–10% risk	1	1	3	4	9
≥10% risk	0	1	0	1	2
Total no.†	2	4	7	10	23
Patients who were alive, no.					
<2% risk	210	41	6	0	257
2–5% risk	136	63	29	10	238
5–10% risk	32	45	17	11	105
≥10% risk	6	8	11	12	37
Total no.†	384	157	63	33	637

* The established model was sPESI (simplified Pulmonary Embolism Severity Index) as a continuous variable. The sPESI incorporates age >80 years, history of malignancy, chronic cardiopulmonary disease, heart rate ≥110 beats/minute, systolic blood pressure <100 mmHg and oxyhemoglobin saturation <90%. Both day-1 serum sodium and bicarbonate were labelled as continuous variables. The net reclassification improvement was estimated at 0.613 (P = 0.0007). The event NRI was 0.39 and the non-event NRI was 0.22.

^†^ The total number of patients (n = 660) included in the reclassification analysis did not match the total derivation cohort (n = 693) due to missing day-1 serum sodium and bicarbonate data for 33 patients.

Based on ROC curve analyses, sodium <135 mmol/L and bicarbonate <23 mmol/L provided the best sensitivity and specificity for predicting in-hospital mortality. Incorporating dichotomized sodium and bicarbonate values to the sPESI led to a NRI for the derivation cohort estimated at 0.483 (P = 0.008) and an IDI of 0.071 (P = 0.0009) (Table D in S1 File).

A sPESI >0 had a sensitivity, specificity, positive predictive value, and negative predictive value of 92.0%, 40.9%, 5.5%, and 99.3% respectively for predicting in-hospital death in the derivation cohort. The corresponding values were 100%, 27.0%, 5.0%, and 100% for the new model (sPESI >0 or sodium <135 mmol/L or bicarbonate <23 mmol/L) for predicting in-hospital death.

### Decision curve analysis

**Fig 2:**
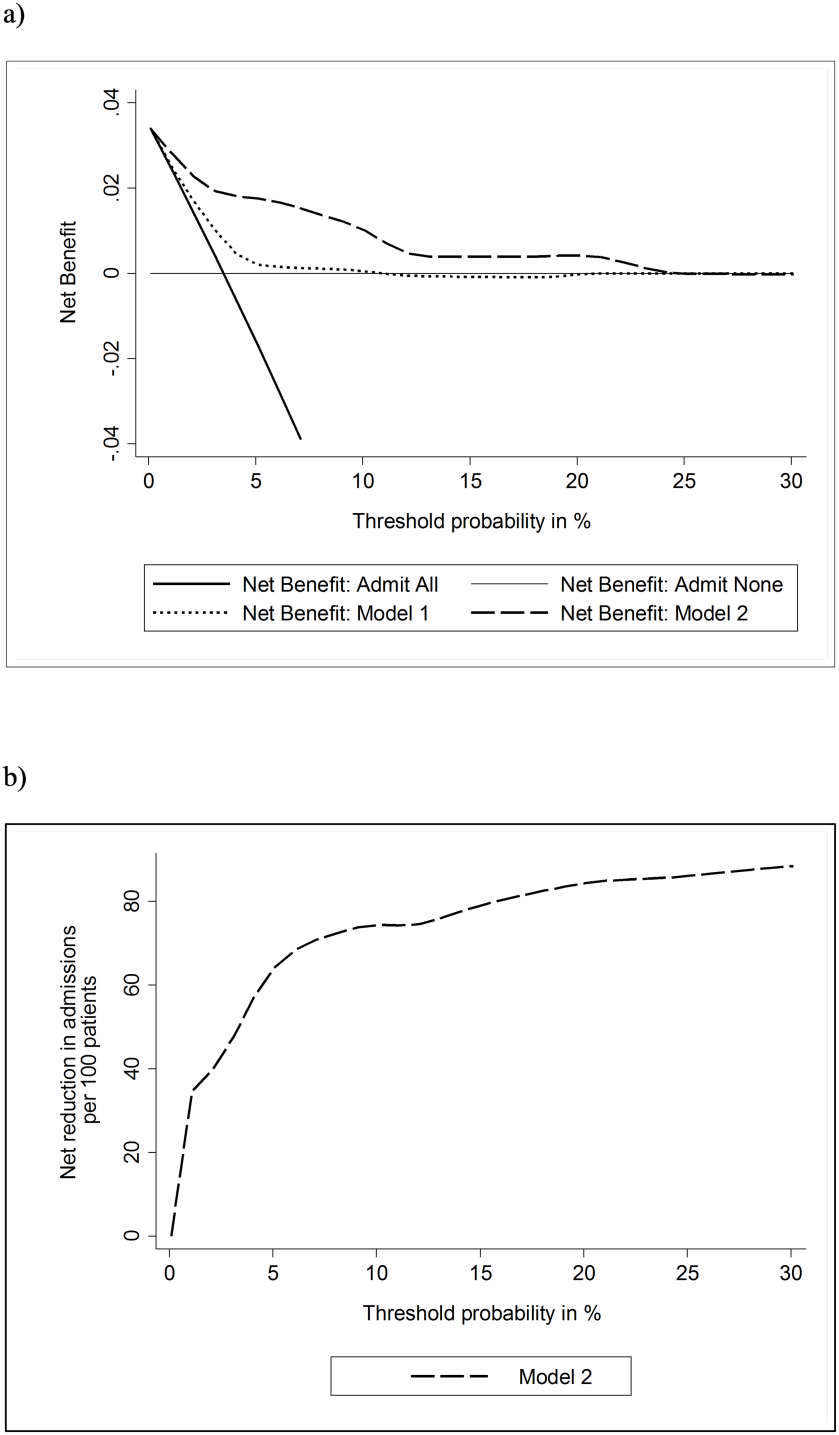
Decision curve analysis and predicted impact on admissions resulting from model 2 to guide clinical management. Net clinical benefit of each of the models across a range of threshold levels of risk of in-hospital death (a). Net reduction in admissions as a result of the use of model 2 to guide clinical management compared to admitting all patients with acute PE (b). Model 1 represents sPESI, model 2 represents sPESI + Na + HCO3. sPESI, simplified Pulmonary Embolism Severity Index; Na, serum sodium; HCO3, serum bicarbonate.

A decision curve analysis compared the clinical utility of the clinical risk prediction models: model 1 (sPESI) and model 2 (sPESI + sodium + bicarbonate). Fig 2a illustrates the net clinical benefit of using the models to risk stratify patients (y-axis) over varying thresholds of risk for in-hospital death (x-axis), relative to assuming no patients will die in-hospital (admit none) and assuming all patients will die (admit all). The decision curve analysis demonstrated that model 2 provided a superior net clinical benefit compared to sPESI across a large range of in-hospital death risk. Fig 2b demonstrates the reduction in unnecessary admissions resulting from the use of model 2 to guide clinical decisions.

### Imputation and population-linkage analyses

Of the 693 patients that formed the derivation cohort, sodium and bicarbonate were not recorded in 30 (4.3%) and 29 (4.2%) patients respectively. In addition, data on admission systolic blood pressure, heart rate, and oxyhemoglobin saturation were missing in 63 (9.1%), 63 (9.1%), and 83 (12.0%) patients respectively. Using multiple imputations method to account for missing data, with the variables in Table 2, multivariable logistic regression analysis of the derivation cohort showed comparable odds ratios for sPESI (OR per-1-point, 2.03; 95% CI 1.86–2.22), sodium (OR per-1 mmol/L increase, 0.84; 95% CI 0.83–0.86), and bicarbonate (OR per-1 mmol/L increase, 0.88; 95% CI 0.86–0.90) for predicting in-hospital death (Table E in S1 File). Using the imputed data, model 2 yielded similar improvements in the AUC for predicting in-hospital death, raising the C-statistic from 0.73 (95% CI 0.71–0.75) for model 1 to 0.87 (95% CI 0.85–0.88) for model 2 (P<0.0001) (Fig A and Table F in S1 File).

There were a total of 36,195 patients (excluding the Concord Hospital cohort) admitted with acute PE state-wide during the study period. There was no difference in the age and sex-adjusted survival after presentation with acute PE between the Concord Hospital cohort and the rest of state-wide cohort (OR 1.04, 95% CI 0.96–1.14, P = 0.34) (Fig B in S1 File) suggesting that the Concord Hospital cohort was representative of the state-wide PE cohort.

### Performance of models in predicting 30-day mortality post-acute PE

Amongst the total 1,426 PE patients, 65 (4.6%) died within 30 days of admission. The independent predictors of 30-day mortality were sPESI (OR per-1-point, 1.97; 95% CI 1.52–2.55), sodium (OR per-1 mmol/L increase, 0.86; 95% CI 0.82–0.91), and bicarbonate (OR per-1 mmol/L increase, 0.90; 95% CI 0.83–0.97). The AUC for sPESI for predicting 30-day mortality was 0.74 (95% CI 0.69–0.79) which increased to 0.83 (95% CI 0.78–0.88) with the addition of sodium and bicarbonate (P = 0.002).

## Discussion

The present study has derived and internally validated a novel risk prediction model for in-hospital death in patients admitted with predominantly sub-massive acute PE by incorporating admission sodium and bicarbonate levels with the sPESI. To our knowledge, this is the first model shown to predict in-hospital death after acute PE. Our model performed significantly better than the sPESI alone in predicting in-hospital death and is capable of reclassifying patients to a more accurate level of risk. The model performed equally well in our validation cohort, giving an unbiased estimate of the predictive capacity of the new model.

Clinical factors such as demographic characteristics and comorbidities have been used to create risk prediction scores such as the PESI [6] and sPESI [7]. However, the use of biomarkers in acute PE for risk stratification is not routine. We and others have shown the importance of a range of biomarkers including serum sodium and arterial base deficit in predicting prognosis after acute PE [8–10]. During an acute PE, low bicarbonate may occur as a result of metabolic acidosis from systemic hypoperfusion, and from metabolic compensation following respiratory alkalosis arising from hyperventilation as a response to hypoxia [19, 20]. The mechanism behind low sodium in patients with PE is not well understood and may reflect neurohormonal activation similar to the mechanisms operational in heart failure and pulmonary hypertensions [21, 22]. The acute deleterious impact of PE on the right ventricle may thus similarly contribute to the lowering of serum sodium. Hormones such as vasopressin are likely to play a role in the development of hyponatremia [23], though the mechanism remains poorly understood.

We hypothesized that the addition of the biomarkers serum sodium and bicarbonate, two commonly measured parameters, to the sPESI would better predict acute mortality after PE compared to the sPESI alone. In the present study, the addition of sodium and bicarbonate to the sPESI significantly improved the ROC-derived C-statistic to greater than 0.8 for predicting in-hospital death.

The C-statistic for sPESI in predicting 30-day mortality was 0.74 in the present study which is comparable to that reported by Jimenez et al [7]. While originally conceived as a predictor of 30-day mortality, we demonstrate that the sPESI can also predict in-hospital mortality. Despite the high sensitivity of the sPESI in predicting in-hospital mortality, our derived model has a higher sensitivity and is able to reclassify low risk patients appropriately to lower levels of risk and identify patients at the highest risk of in-hospital death compared to sPESI alone.

### Implications of model on clinical practice

When faced with a patient with acute pulmonary embolism, one of the pertinent decisions is whether the patient warrants admission for monitoring and commencement of anticoagulation. Our risk prediction model may allow more appropriate allocation of resources, facilitating earlier discharge of the lowest risk patients for outpatient management. The identification of patients who are at very low risk of early mortality may lead to health resource savings and more appropriate inpatient resource allocation to higher risk patients. The availability of direct-acting anticoagulants coupled with accurate risk stratification of patients presenting to hospital with acute PE may have a significant impact on workflow with substantial cost savings. The ability of this model to facilitate safe early discharge and outpatient management of low risk patients will need assessment in a prospective study.

Our decision curve analysis illustrates the clinical benefit of the new model over a range of in-hospital mortality risk thresholds. If the threshold risk for in-hospital death before a clinician would admit a patient ranged between 1% and 5%, decisions based on our derived model would result in greater net clinical benefit (un-necessary admission of a patient who would not die in hospital) than either the sPESI alone or the clinical practice of admitting all patients. If, for example, a clinician uses a threshold risk of 2% for in-hospital death before deciding to admit a patient with acute PE, the use of our model to guide clinical decisions would result in 43 fewer admissions per 100 presentations without discharging any patients who would have otherwise died in-hospital. The corresponding net reduction in admissions at threshold risks of 1% and 5% are 28 and 65 per 100 presentations respectively.

### Limitations

The main limitation of our study is its retrospective single-center design, and thus our findings will need external validation. It is notable the rate of in-hospital death in our cohort is relatively low compared to other cohorts that have been reported previously [5, 24]. By using a population-linkage method, we demonstrated that the age and sex-adjusted mortality outcome of our study cohort was similar to the rest of the state-wide PE cohort. Though the derivation and validation cohorts were not chosen at random, we believe this selection method minimized the potential impact of any improvements in PE management over time. Although there were some missing data, these were small and we showed that our findings were not altered when a multiple imputations method was utilized.

Biomarkers such as troponin and brain natriuretic peptide have also been shown to be important in risk stratification of pulmonary embolism [25]. These biomarkers were not included in our model as these parameters were not routinely assessed in the majority of patients in our cohort. Additionally, the presence of ECG changes or evidence of right ventricular strain on imaging was not assessed in this study and the utility of these clinical findings as additions to our model is an area for future exploration. Finally, as our study cohorts were comprised of patients who were admitted to hospital with acute PE, the results of our study may not be generalizable to patients with PE who are diagnosed and managed in the community.

## Conclusion

Despite optimal medical therapy, PE continues to be an important cause of acute death. The addition of readily available biomarkers, namely serum sodium and bicarbonate, to the sPESI, accurately predicts in-hospital mortality after acute PE. An accurate risk prediction model can help identify patients who may benefit from less or more intensive therapy after presentation with acute PE and facilitate approporiate allocation of health care resources.

## Supporting information

S1 File **Text A:** Variables collected in the PE registry. **Table A:** Characteristics of patients stratified by day-1 serum sodium level (derivation cohort). **Table B:** Characteristics of patients stratified by day-1 serum bicarbonate (derivation cohort). **Table C:** Risk prediction modelling for in-hospital death after acute PE. **Table D:** Reclassification of patients based on dichotomized serum sodium and bicarbonate on admission (derivation cohort). **Table E:** Predictors of in-hospital mortality after acute PE based on 20 imputations for missing data (derivation cohort). **Table F:** Risk prediction modelling for in-hospital death after acute PE based on derivation cohort with imputed missing data. **Fig A:** Receiver operating characteristic curves in imputed derivation cohort (a, b) and impact of adding serum sodium and bicarbonate to sPESI for prediction of in-hospital mortality after presentation with acute PE. **Fig B:** Comparison of age and sex-adjusted survival after presentation with acute PE between Concord cohort and the state-wide (New South Wales) cohort.
(DOCX)

## Abbreviations

AUC: area under curve
CI: confidence interval
CVD: cardiovascular disease
CRD: chronic respiratory disease
DVT: deep vein thrombosis
eGFR: estimated glomerular filtration rate
HCO3: serum bicarbonate
Na: serum sodium
PE: pulmonary embolism
PESI: pulmonary embolism severity index
ROC: receiver operating characteristic
sPESI: simplified pulmonary embolism severity index
