# Supplementary material for: Predicting in-hospital death during acute presentation with pulmonary embolism to facilitate early discharge and outpatient management
Source: PLoS One. 2017 Jul 13;12(7):e0179755. doi: 10.1371/journal.pone.0179755 (PMC5509112; doi:10.1371/journal.pone.0179755)
Supplement: S1 File — Text A: Variables collected in the PE registry. Table A: Characteristics of patients stratified by day-1 serum sodium level (derivation cohort). Table B: Characteristics of patients stratified by day-1 serum bicarbonate (derivation cohort). Table C: Risk prediction modelling for in-hospital death after acute PE. Table D: Reclassification of patients based on dichotomized serum sodium and bicarbonate on admission (derivation cohort). Table E: Predictors of in-hospital mortality after acute PE based on 20 imputations for missing data (derivation cohort). Table F: Risk prediction modelling for in-hospital death after acute PE based on derivation cohort with imputed missing data. Fig A: Receiver operating characteristic curves in imputed derivation cohort (a, b) and impact of adding serum sodium and bicarbonate to sPESI for prediction of in-hospital mortality after presentation with acute PE. Fig B: Comparison of age and sex-adjusted survival after presentation with acute PE between Concord cohort and the state-wide (New South Wales) cohort. (DOCX) [file pone.0179755.s001.docx]

**Supporting information: S1 File**

**S1 File: Text A:** Variables collected in the PE registry. **Table A:** Characteristics of patients stratified by day-1 serum sodium level (derivation cohort). **Table B:** Characteristics of patients stratified by day-1 serum bicarbonate (derivation cohort). **Table C:** Risk prediction modelling for in-hospital death after acute PE. **Table D:** Reclassification of patients based on dichotomized serum sodium and bicarbonate on admission (derivation cohort). **Table E:** Predictors of in-hospital mortality after acute PE based on 20 imputations for missing data (derivation cohort). **Table F:** Risk prediction modelling for in-hospital death after acute PE based on derivation cohort with imputed missing data. **Figure A:** Receiver operating characteristic curves in imputed derivation cohort (a, b) and impact of adding serum sodium and bicarbonate to sPESI for prediction of in-hospital mortality after presentation with acute PE. **Figure B:** Comparison of age and sex-adjusted survival after presentation with acute PE between Concord cohort and the state-wide (New South Wales) cohort.

**Text A: Variables collected in the PE registry**

Details of patient’s admission collected included: symptoms, hemodynamic profile (admission blood pressure, heart rate and oxyhaemoglobin saturation), the type of imaging modality used to diagnose the PE, length of admission, blood profiles at admission (including day-1 serum sodium, bicarbonate, and estimated glomerular filtration rate), and in-hospital outcomes.

In addition, details of comorbidities present at the time of admission were also collected, and included: history of cardiovascular disease (including atrial fibrillation/flutter, ischemic heart disease, previous coronary artery bypass graft surgery or percutaneous coronary intervention, heart failure, valvular heart disease, peripheral vascular disease, stroke and prosthetic heart valves), cardiac risk factors (hypertension, hyperlipidaemia, diabetes, current or ex-smoking), malignancy, chronic respiratory disease (asthma and/or emphysema), obstructive sleep apnoea, neurodegenerative disease (dementia and/or Parkinson’s disease) and chronic kidney disease.

Description of the variables collected in the PE registry.

PE, pulmonary embolism;

| **Table A: Characteristics of patients stratified by day-1 serum sodium level (derivation cohort)**^*^ | | | |
| --- | --- | --- | --- |
| **Characteristic** | **Na<135 mmol/L (n=87)** | **Na≥135 mmol/L**  **(n=576)** | **P value** |
| Age, year | 72.0 ± 14.2 | 66.8 ± 16.7 | 0.006 |
| Male, no. (%) | 45 (51.7) | 255 (44.3) | 0.21 |
| CVD, no. (%) | 45 (51.7) | 217 (37.7) | 0.01 |
| Peripheral vascular disease, no. (%) | 12 (13.8) | 57 (9.9) | 0.26 |
| Stroke, no. (%) | 7 (8.0) | 13 (2.3) | 0.01 |
| Hypertension, no. (%) | 25 (28.7) | 148 (25.7) | 0.60 |
| Diabetes, no. (%) | 14 (16.1) | 74 (12.8) | 0.40 |
| Dyslipidaemia, no. (%) | 14 (16.1) | 59 (10.2) | 0.14 |
| Current smoking, no. (%) | 6 (6.9) | 50 (8.7) | 0.68 |
| CRD, no. (%) | 10 (11.5) | 65 (11.3) | 1.00 |
| Pulmonary hypertension, no. (%) | 0 (0) | 10 (1.7) | 0.38 |
| DVT during admission, no. (%) | 10 (11.5) | 113 (19.6) | 0.08 |
| Malignancy, no. (%) | 33 (37.9) | 113 (19.6) | <0.001 |
| Chronic kidney disease, no. (%) | 4 (4.6) | 37 (6.4) | 0.64 |
| **Hemodynamic and biochemistry parameters on admission** | | | |
| Systolic blood pressure, mmHg† | 135.9 ± 23.6 | 141.2 ± 24.3 | 0.08 |
| Heart rate, beats/min‡ | 93.7 ± 23.6 | 87.2 ± 20.9 | 0.02 |
| Oxyhaemoglobin saturation, %§ | 94.8 ± 5.2 | 95.6 ± 3.6 | 0.07 |
| sPESIǁ | 1.3 ± 1.0 | 0.8 ± 0.9 | <0.001 |
| Day-1 HCO3<24mmol/L, no. (%) | 52 (59.8) | 215/573 (37.5) | <0.001 |
| Day-1 HCO3, mmol/L | 23.4 ± 4.2 | 24.7 ± 3.6 | 0.002 |
| Day-1 eGFR, mL/min/1.73m^2^¶ | 80.9 ± 38.5 | 78.4 ± 34.3 | 0.55 |
| **Outcome** | | | |
| In-hospital death, no. (%) | 13 (14.9) | 10 (1.7) | <0.001 |

* Plus-minus values are means ± standard deviation.

† Number of patients with admission systolic blood pressure recorded in the Na<135 mmol/L and Na≥135 mmol/L cohorts were 71/87 and 530/576 respectively.

‡ Number of patients with admission heart rate recorded in the Na<135 mmol/L and Na≥135 mmol/L cohorts were 71/87 and 530/576 respectively.

§ Number of patients with admission oxyhaemoglobin saturations recorded in the Na<135 mmol/L and Na≥135 mmol/L cohorts were 69/87 and 518/576 respectively.

ǁ Number of patients with sPESI calculated in the Na<135 mmol/L and Na≥135 mmol/L cohorts were 69/87 and 518/576 respectively.

¶ Number of patients with admission eGFR recorded in the Na<135 mmol/L and Na≥135 mmol/L cohorts were 87/87 and 575/576 respectively.

CVD, cardiovascular disease (included coronary artery disease, heart failure, valvular heart disease and arrhythmias); CRD, chronic respiratory disease (included asthma, chronic obstructive pulmonary disease and interstitial lung disease); DVT, deep vein thrombosis; sPESI, simplified Pulmonary Embolism Severity Index; Na, serum sodium; HCO3, serum bicarbonate; eGFR, estimated glomerular filtration rate.

The sPESI incorporates age >80 years, history of malignancy, chronic cardiopulmonary disease, heart rate ≥110 beats/minute, systolic blood pressure <100 mmHg and oxyhaemoglobin saturation <90%.

| **Table B: Characteristics of patients stratified by day-1 serum bicarbonate (derivation cohort)**^*^ | | | |
| --- | --- | --- | --- |
| **Characteristic** | **HCO3<24 mmol/L (n=267)** | **HCO3≥24 mmol/L**  **(n=397)** | **P value** |
| Age, year | 67.5 ± 16.9 | 67.5 ± 16.2 | 0.99 |
| Male, no. (%) | 116 (43.4) | 184 (46.3) | 0.48 |
| CVD, no. (%) | 110 (41.2) | 154 (38.8) | 0.57 |
| Peripheral vascular disease, no. (%) | 26 (9.7) | 44 (11.1) | 0.61 |
| Stroke, no. (%) | 10 (3.7) | 10 (2.5) | 0.37 |
| Hypertension, no. (%) | 78 (29.2) | 95 (23.9) | 0.15 |
| Diabetes, no. (%) | 37 (13.9) | 51 (12.8) | 0.73 |
| Dyslipidaemia, no. (%) | 34 (12.7) | 41 (10.3) | 0.38 |
| Current smoking, no. (%) | 18 (6.7) | 38 (9.6) | 0.25 |
| CRD, no. (%) | 30 (11.2) | 46 (11.6) | 1.00 |
| Pulmonary hypertension, no. (%) | 1 (0.4) | 9 (2.3) | 0.06 |
| DVT during admission, no. (%) | 40 (15.0) | 83 (20.9) | 0.07 |
| Malignancy, no. (%) | 53 (19.9) | 93 (23.4) | 0.29 |
| Chronic kidney disease, no. (%) | 22 (8.2) | 20 (5.0) | 0.11 |
| **Hemodynamic and biochemistry parameters on admission** | | | |
| Systolic blood pressure, mmHg† | 140.9 ± 24.9 | 140.2 ± 23.9 | 0.74 |
| Heart rate, beats/min‡ | 90.0 ± 22.5 | 86.5 ± 20.3 | 0.05 |
| Oxyhaemoglobin saturation, %§ | 95.7 ± 3.6 | 95.5 ± 3.9 | 0.44 |
| sPESIǁ | 0.9 ± 0.9 | 0.9 ± 0.9 | 0.96 |
| Day-1 Na<135mmol/L, no. (%) | 52 (19.5) | 35/393 (8.9) | <0.001 |
| Day-1 Na, mmol/L | 137.8 ± 4.0 | 139.1 ± 3.8 | <0.001 |
| Day-1 eGFR, mL/min/1.73m^2^¶ | 76.7 ± 38.8 | 80.1 ± 32.0 | 0.22 |
| **Outcome** | | | |
| In-hospital death, no. (%) | 14 (5.2) | 10 (2.5) | 0.09 |

* Plus-minus values are means ± standard deviation.

† Number of patients with admission systolic blood pressure recorded in the HCO3<24 mmol/L and HCO3≥24 mmol/L cohorts was 239/267 and 363/397 respectively.

‡ Number of patients with admission heart rate recorded in the HCO3<24 mmol/L and HCO3≥24 mmol/L cohorts was 239/267 and 363/397 respectively.

§ Number of patients with admission oxyhaemoglobin saturations recorded in the HCO3<24 mmol/L and HCO3≥24 mmol/L cohorts was 231/267 and 357/397 respectively.

ǁ Number of patients with sPESI calculated in the HCO3<24 mmol/L and HCO3≥24 mmol/L cohorts was 231/267 and 357/397 respectively.

¶ Number of patients with admission eGFR recorded in the HCO3<24 mmol/L and HCO3≥24 mmol/L cohorts was 267/267 and 392/397 respectively

CVD, cardiovascular disease (included coronary artery disease, heart failure, valvular heart disease and arrhythmias); CRD, chronic respiratory disease (included asthma, chronic obstructive pulmonary disease and interstitial lung disease); DVT, deep vein thrombosis; sPESI, simplified Pulmonary Embolism Severity Index; Na, serum sodium; HCO3, serum bicarbonate; eGFR, estimated glomerular filtration rate.

The sPESI incorporates age >80 years, history of malignancy, chronic cardiopulmonary disease, heart rate ≥110 beats/minute, systolic blood pressure <100 mmHg and oxyhaemoglobin saturation <90%.

| **Table C: Risk prediction modelling for in-hospital death after acute PE** | |
| --- | --- |
| **Risk models** | **C-statistic (95% CI)** |
| Derivation cohort - Model 1* | 0.71 (0.62 – 0.80) |
| Derivation cohort - Model 2* | 0.86 (0.79 – 0.93) |
| Validation cohort - Model 2 | 0.85 (0.78 – 0.92) |

Derivation cohort - Model 1 - sPESI; the Hosmer-Lemeshow 𝜒^2^ statistic was 3.62, P=0.16.

Derivation cohort - Model 2 - sPESI + Na + HCO3; the Hosmer-Lemeshow 𝜒^2^ statistic was 6.80, P=0.56.

Validation cohort - Model 2 - sPESI + Na + HCO3; the Hosmer-Lemeshow 𝜒^2^ statistic was 5.57, P=0.70.

* Model 1 vs Model 2 in derivation cohort, P=0.001.

Unstandardized regression co-efficients:

Derivation cohort – Model 1: constant -4.15, sPESI 0.72.

Derivation cohort – Model 2: constant 24.83, sPESI 0.56, Na -0.19, HCO3 -0.14.

Validation cohort – Model 2: constant 17.02, sPESI 0.59, Na -0.13, HCO3 -0.16.

CI, confidence interval; Na, serum sodium; HCO3, serum bicarbonate; sPESI, simplified Pulmonary Embolism Severity Index.

The sPESI incorporates age >80 years, history of malignancy, chronic cardiopulmonary disease, heart rate ≥110 beats/minute, systolic blood pressure <100 mmHg and oxyhaemoglobin saturation <90%.

| **Table D: Reclassification of patients based on dichotomized serum sodium and bicarbonate on admission (derivation cohort)** | | | | | |
| --- | --- | --- | --- | --- | --- |
| **Established model - sPESI^*^** | **sPESI + day-1 serum sodium and bicarbonate^*^** | | | | |
|  | <2% risk | 2-5% risk | 5-10% risk | ≥10% risk | Total no. |
| Patients who died, no. |  | | | | |
| <2% risk | 0 | 0 | 1 | 1 | 2 |
| 2-5% risk | 3 | 2 | 1 | 4 | 10 |
| 5-10% risk | 1 | 0 | 3 | 5 | 9 |
| ≥10% risk | 0 | 1 | 0 | 1 | 2 |
| Total no.† | 4 | 3 | 5 | 11 | 23 |
| Patients who were alive, no. |  |  |  |  |  |
| <2% risk | 176 | 67 | 8 | 6 | 257 |
| 2-5% risk | 150 | 52 | 25 | 11 | 238 |
| 5-10% risk | 68 | 0 | 24 | 13 | 105 |
| ≥10% risk | 0 | 18 | 8 | 11 | 37 |
| Total no.† | 394 | 137 | 65 | 41 | 637 |

* The established model was sPESI (simplified Pulmonary Embolism Severity Index) as a continuous variable. The sPESI incorporates age >80 years, history of malignancy, chronic cardiopulmonary disease, heart rate ≥110 beats/minute, systolic blood pressure <100 mmHg and oxyhaemoglobin saturation <90%. Day-1 serum sodium and bicarbonate were labelled as dichotomous variables (< or ≥ 135 mmol/L and < or ≥ 23 mmol/L respectively). The net reclassification improvement was estimated at 0.483 (P=0.008). Addition of serum sodium <135 mmol/L and serum bicarbonate <23 mmol/L to the sPESI increased the area under the ROC curve for predicting in-hospital death from 0.71 (95% CI 0.62 – 0.80) to 0.83 (95% CI 0.75 – 0.91) (P=0.02).

† The total number of patients (n=660) included in the reclassification analysis did not match the total derivation cohort (n=693) due to missing day-1 serum sodium and bicarbonate data for 33 patients.

| **Table E: Predictors of in-hospital mortality after acute PE based on 20 imputations for missing data (derivation cohort)** | | |
| --- | --- | --- |
| **Variables** | **Odds ratio (95% CI)** | **P value** |
| **Multivariable analysis** | | |
| sPESI - per 1-point | 2.03 (1.86 – 2.22) | <0.001 |
| Day-1 Na - per 1mmol/L increase | 0.84 (0.83 – 0.86) | <0.001 |
| Day-1 HCO3 - per 1mmol/L increase | 0.88 (0.86 – 0.90) | <0.001 |

CI, confidence interval; Na, serum sodium; HCO3, serum bicarbonate; sPESI, simplified Pulmonary Embolism Severity Index.

The sPESI incorporates age >80 years, history of malignancy, chronic cardiopulmonary disease, heart rate ≥110 beats/minute, systolic blood pressure <100 mmHg and oxyhaemoglobin saturation <90%.

| **Table F: Risk prediction modelling for in-hospital death after acute PE based on derivation cohort with imputed missing data^*^** | |
| --- | --- |
| **Risk models** | **C-statistic (95% CI)** |
| Imputed derivation cohort - Model 1† | 0.73 (0.71 – 0.75) |
| Imputed derivation cohort - Model 2† | 0.87 (0.85 – 0.88) |

* 20 imputations used

Model 1 - sPESI

Model 2 - sPESI + Na + HCO3

† Model 1 vs Model 2 in derivation cohort, P<0.001.

Unstandardized regression co-efficients:

Imputed derivation cohort – Model 1: constant -4.40, sPESI 0.77.

Imputed derivation cohort – Model 2: constant 23.56, sPESI 0.71, Na -0.17, HCO3 -0.13.

CI, confidence interval; Na, serum sodium; HCO3, serum bicarbonate; sPESI, simplified Pulmonary Embolism Severity Index.

The sPESI (simplified Pulmonary Embolism Severity Index) incorporates age >80 years, history of malignancy, chronic cardiopulmonary disease, heart rate ≥110 beats/minute, systolic blood pressure <100 mmHg and oxyhaemoglobin saturation <90%.

**Figure legends:**

**Figure A: Receiver operating characteristic curves in imputed derivation cohort (a, b) and impact of adding serum sodium and bicarbonate to sPESI for prediction of in-hospital mortality after presentation with acute PE.**

The area under the ROC curve (AUC) for sPESI for predicting in-hospital death in the imputed derivation cohort was 0.73 (95% CI 0.71 – 0.75). The AUC for the new model including sPESI + day-1 Na + day-1 HCO3 for predicting in-hospital death in the imputed derivation cohort was 0.87 (95% CI 0.85 – 0.88).

sPESI, simplified Pulmonary Embolism Severity Index; Na, serum sodium; HCO3, serum bicarbonate; ROC, receiver operating characteristics. The sPESI incorporates age >80 years, history of malignancy, chronic cardiopulmonary disease, heart rate ≥110 beats/minute, systolic blood pressure <100 mmHg and oxyhaemoglobin saturation <90%.

**Figure B: Comparison of age and sex-adjusted survival after presentation with acute PE between Concord cohort and the state-wide (New South Wales) cohort.**

The solid line represents the PE cohort from the state of New South Wales (NSW) (not including the Concord cohort), while the broken line represents the Concord PE cohort. There was no mortality difference after acute PE between the Concord cohort and the state-wide PE cohort (OR 1.04, 95% CI 0.96 – 1.14, P=0.34).

**Figure A: Receiver operating characteristic curves in imputed derivation cohort (a, b) and impact of adding serum sodium and bicarbonate to sPESI for prediction of in-hospital mortality after presentation with acute PE.**

1. Model 1 - sPESI


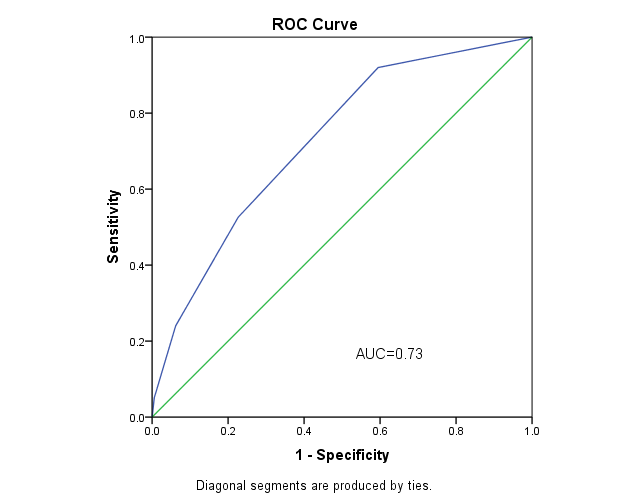


1. Model 2 - sPESI + Na + HCO3


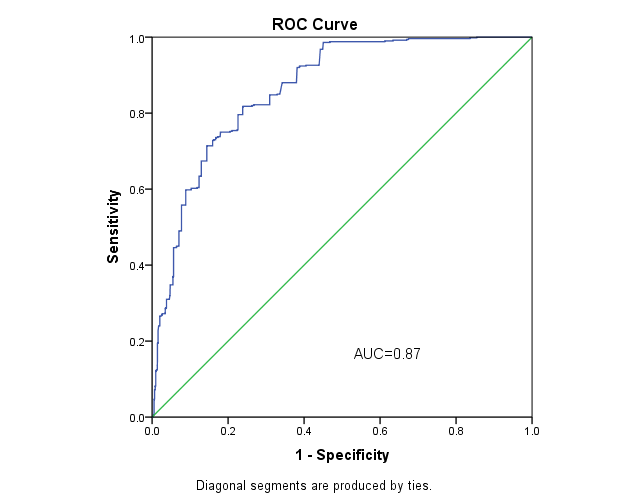


**Figure B: Comparison of age and sex-adjusted survival after presentation with acute PE between Concord cohort and the rest of state-wide (New South Wales) cohort.**

**
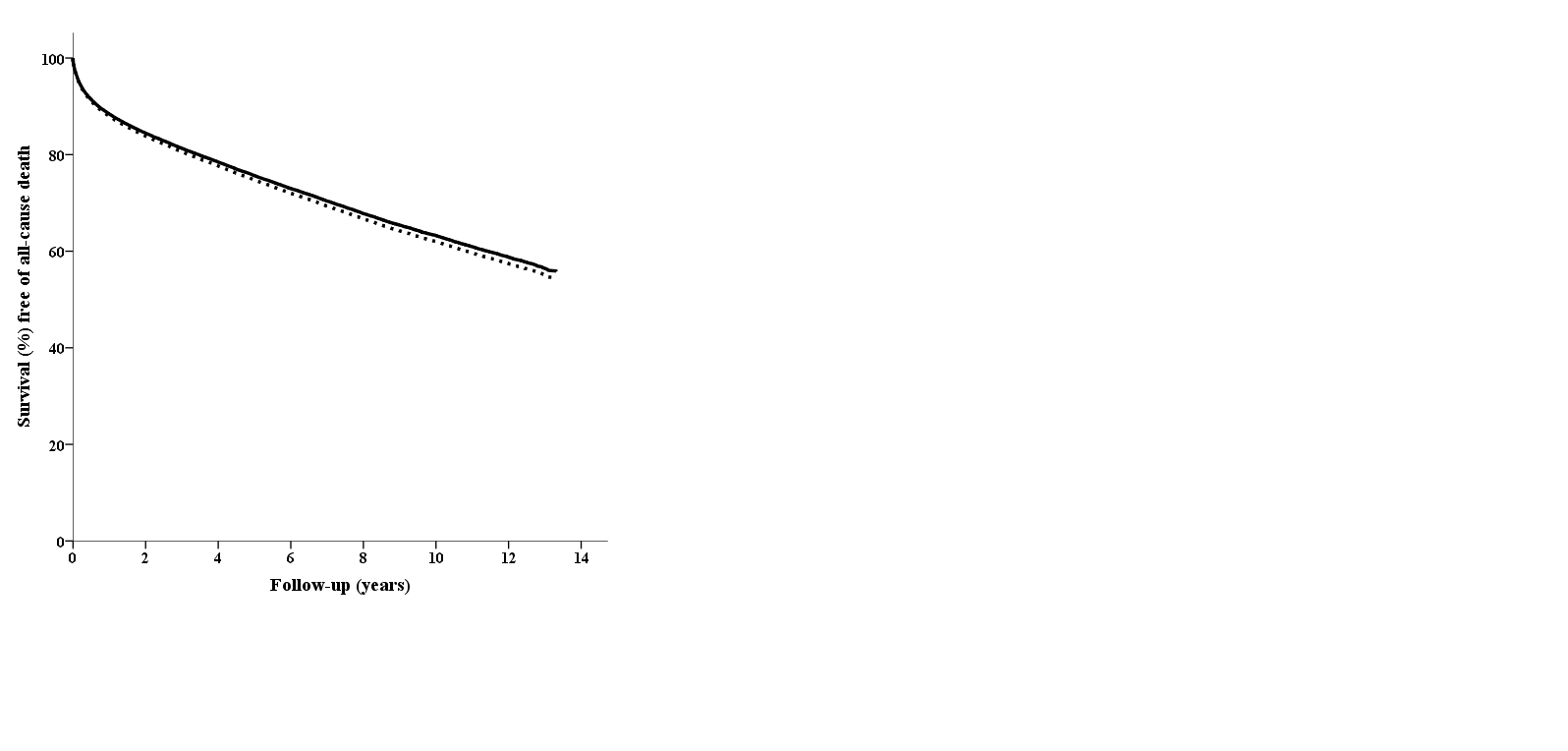
**

State-wide PE cohort

Concord PE cohort
